# Can Morning Light Phase Advance Human Melatonin Rhythms in Less Than 24 h?

**DOI:** 10.1111/jpi.70134

**Published:** 2026-03-15

**Authors:** Clara López‐Velasco, Carolin Franziska Reichert, Christian Cajochen

**Affiliations:** ^1^ Centre for Chronobiology Psychiatric Hospital of the University of Basel Basel Switzerland; ^2^ Research Cluster Molecular and Cognitive Neurosciences, Department of Biomedicine University of Basel Basel Switzerland; ^3^ Present address: MRC/CSO Social &Public Health Sciences Unit University of Glasgow United Kingdom

**Keywords:** circadian rhythm, DLMO, light, melanopic EDI, melatonin, phase advance, phase response curve

## Abstract

Light is the primary cue that synchronises the human circadian system to the 24‐h day, advancing or delaying circadian rhythms depending on its timing. While it is known that morning light induces phase advances, most studies assess the timing of dim‐light melatonin onset (DLMO) in the subsequent circadian cycle, over 24 h after the light intervention. However, it is unclear whether these phase advances occur within the same circadian cycle as the light intervention or the next one. This narrative review addresses the question of whether morning light can phase‐advance human melatonin rhythms in less than 24 h. To answer this question, we review studies that use same‐day or single‐cycle protocols, in which light exposure and post‐intervention DLMO assessment occur within the same 24‐h period. To compare light interventions across studies, melanopic equivalent daylight illuminance (mEDI) values were estimated and related to the magnitude of the observed phase advance. The reviewed research suggests that modest phase advances of 10–30 min can be achieved within the same circadian cycle if light is delivered shortly after waking up in the morning. This is particularly effective if the light is bright, blue‐enriched, or if it is delivered for a long time (over 1 h). There was a statistical trend (*r* = 0.51, *p* = 0.06) towards a positive association between the mEDI of the light intervention and the magnitude of the phase advance. Overall, same‐day phase‐advances seem possible but not well characterised, and more targeted work is needed to determine whether morning light can phase‐advance human melatonin rhythms in less than 24 h. If this is confirmed, the length of circadian protocols could be reduced, accelerating the clinical use of treatments for circadian rhythms sleep–wake disorder.

## 1 Introduction

**Figure 1:**
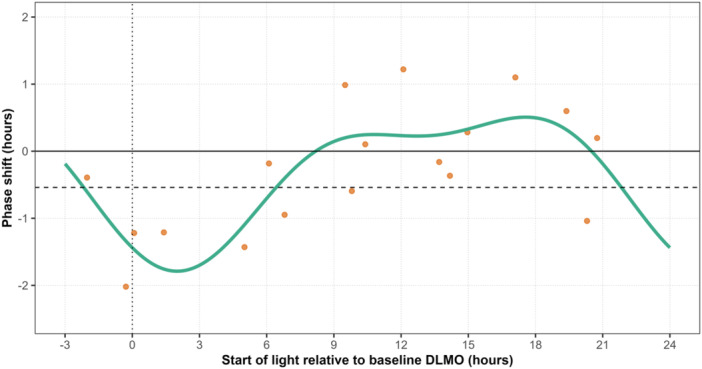
Phase response curve (PRC) for dim‐light melatonin onset (DLMO) in response to light exposure. Phase shifts in response to a 1‐h bright light pulse were extracted from St. Hilaire et al. [7, Figure 3B] using WebPlotDigitizer [8] and modelled using a two‐harmonic curve fit (*n* = 18). Phase shifts are plotted relative to light exposure onset, with positive values indicating phase advances and negative values indicating phase delays. The dotted vertical line marks the timing of DLMO. The dashed horizontal line represents the line of no net phase shift (−0.54 h), corresponding to the expected circadian drift based on an intrinsic average period of 24.18 h [9]. Phase shifts were measured over three 24‐h cycles in dim background light (< 3 lx). The data show a transition from delays to advances ~6 h after DLMO, and from advances to delays ~2 h before DLMO.

Light serves as the vital environmental cue that synchronises the human circadian timing system to the 24‐h day. This process is called circadian entrainment and involves adjusting the timing of circadian rhythms through phase shifts [1]. However, it also involves the biological clock gradually adjusting its period to align with the 24‐h day, meaning less correction is required over time [2]. By aligning the internal biological clock with the external world (i.e. circadian entrainment), light plays a crucial role in regulating physiological processes like the sleep‐wake cycle, metabolism, cognition and mood [3, 4]. Depending on its timing, light exposure can either cause phase advances (shifting rhythms earlier) or phase delays (shifting rhythms later) in human circadian rhythms of melatonin, core body temperature, and cortisol [5, 6]. This relationship is commonly represented using a phase response curve (PRC; Figure 1), which maps the direction and magnitude of circadian shifts in response to light administered at different times [10]. Broadly, light exposure at the end of the biological night induces phase advances, while light exposure at the start of the biological night induces phase delays [10].

Phase shifts are typically assessed by comparing the timing of the dim‐light melatonin onset (DLMO) before and after a light stimulus, measured under controlled laboratory conditions. Then, most studies measure the post‐stimulus DLMO on the subsequent circadian cycle (i.e. more than 24 h after light exposure). This raises the question of whether these light‐induced shifts occur within the same circadian cycle (< 24 h), following the light intervention, or only appear in the next cycle.

Answering this question has both methodological and translational relevance. From a research perspective, circadian experiments are costly and time‐intensive, often involving data collection periods spanning over 32 h [11]. This also makes participant recruitment challenging, resulting in more relaxed protocols that fail to control for important factors such as daytime light exposure. If it can be demonstrated that circadian responses to light influence the circadian timing system within the same cycle, research protocols could be shortened. In turn, this would enable researchers to map the effects of light on human physiology in a more time‐ and cost‐effective manner.

From a practical standpoint, clarifying whether phase advances can be achieved within a single circadian cycle would improve the recommendations of light‐based interventions for shift workers, individuals experiencing jet lag, or patients with circadian rhythm sleep–wake disorders. The ability to accurately time light exposure depends on an understanding of the temporal dynamics of light's effects on the circadian system.

Another important observation in circadian research is the asymmetry between phase delays and phase advances, with light producing larger delays more consistently [7]. This can be partly explained by human intrinsic period length [9, 12, 13]. Period length refers to the natural duration of a circadian cycle without external time cues, it is typically longer than a solar day and thus facilitates phase delays. This asymmetry is also evident in the more severe jet‐lag symptoms experienced after eastward travel, which requires a phase advance [14].

Nevertheless, the extent to which circadian phase advances are more difficult to achieve experimentally remains unclear, as do the methodological factors influencing this. For example, some light PRC studies still observe smaller maximal phase advances compared to delays, even when accounting for the average human intrinsic circadian drift [7]. Compensating for the estimated average drift over several circadian cycles adds noise to the observed phase‐shift measures as the contribution of individual period length accumulates. A shorter protocol for investigating the effects of a light intervention would be less affected by this accumulation of individual variability. Another contributing factor might be the sleep deprivation involved in PRC constant routine protocols, which has been found to blunt light‐induced phase advances [15]. Overall, while research has found that light exposure at night can delay human melatonin rhythms within the same circadian cycle [16], same‐cycle phase advances remain under‐explored.

To address these questions, it is important to consider whether circadian phase advances can be reliably induced within the same circadian cycle as the light exposure, rather than only being observable after a full cycle has elapsed. This narrative review, therefore, examines the temporal dynamics of light‐induced circadian phase advances, evaluating whether circadian phase advances can occur within the same circadian cycle. To do this, we review studies that use same‐day or single‐cycle protocols, in which light exposure and post‐intervention DLMO assessment occur within the same 24‐h period.

## 2 Methods

### 2.1 Study Selection

A literature search in the PubMed database was performed in May 2025 using combinations of keywords such as ‘light’, ‘circadian’, ‘melatonin’, ‘phase advance’. Inclusion criteria were:

a. sample of healthy adult participants,
b. study includes at least one manipulation of light intensity, timing, duration, or spectral composition,
c. study reports pre‐ versus post‐light intervention DLMO or reports dim‐light DLMO versus bright light DLMO and
d. phase advances are measured within the same circadian cycle as the light intervention, and sleep deprivation is not involved.

In total, five papers met the inclusion criteria, which together investigated 16 experimental light intervention manipulations.

### 2.2 Light Quantification

Based on changes in reporting standards for light interventions, melanopic equivalent daylight illuminance (mEDI) values were calculated using an Excel worksheet: the ‘Human Centric Lighting Toolkit’, as described previously [17]. This yields identical results to those obtained using the toolkit from the International Commission on Illumination (e.g., CIE S 026). When available, spectral data reported by the authors were used directly. If spectral data were not reported, mEDI was estimated using the closest matching built‐in spectrum provided in the toolbox, based on the described light source and correlated colour temperature (CCT).

Among the reviewed studies, only Ohashi et al. [18] reported mEDI directly. For Gabel et al. [19], spectral composition data were available, resulting in two calculated mEDI values (103.59 and 109.45), which were averaged. For Danilenko et al. [20], mEDI was estimated based on the spectral composition of a halogen lamp with a standard CCT of 3000 K, as only the lamp type was specified. For Kozaki et al. [21, 22], mEDI values were derived using the reported light source type and CCT. For dawn simulation protocols (e.g. [19, 20]), mEDI was estimated based on the maximum light intensity reached during the intervention, rather than the average intensity across the full simulation.

## 3 Results and Discussion

This section examines whether light exposure can induce a measurable advance in circadian phase, measured as the DLMO, within the same 24‐h period as the stimulus. Although relatively few studies have directly tested this, the available evidence suggests that phase advances can occur within the same cycle under specific conditions. The extent of these advances depends on factors including light intensity, timing, duration, spectral composition, and individual variability. Table 1 provides a detailed summary of the studies reviewed under this aim, including the light parameters and phase shift outcomes.

**Table 1:** Summary of studies examining the effects of light exposure on melatonin phase within the same cycle.

	Participant characteristics			Light intervention							Results		
Paper	*n*	Age (SD)	Sex	Source	Characteristics	Timing	Duration	Intensity (lx)	mEDI	Daytime light conditions	DLMO phase advance in minutes (SD)	Post‐light intervention DLMO timing (SD)	Significance
[22]	11	21.8 (3.3)	Male only	Fluorescent	4523 K	1 h after wake	3 h	750	469.18	Uncontrolled	8 (15)	—	ns
								1500	938.37		4 (20)	—	ns
								3000	1876.73		15 (13)	—	*
								6000	3753.47		26 (18)	—	**
								12 000	7506.94		27 (21)	—	**
[21]	10	20.8 (1.1)	Male only	None	—	1 h after wake	3 h	< 10	—	Dim (unspecified intensity)	—	22:15 (69)	—
				Fluorescent	4523 K			100	62.56		—	22:10 (70)	ns
								300	187.67		—	21:58 (72)	ns
								900	563.02		—	21:52 (76)	ns
								2700	1689.06		—	21:51 (57)	ns
[18]	27	22.2 (2.3)	12 male, 15 female	Fluorescent	4103 K	From wake, 11 h after DLMO	1 h	8000	4951	~3 lx	11 (22)	—	*
[20]	8	25.9 (4.6)	Male only	Halogen	—	42 min before wake	2.2 h	0–1000	506.58	< 100 lx	20 (‐)	—	*
	8	25.3 (2.1)				—	—	0–2000	1013.16	< 30 lx	20 (‐)	—	*
[19]	17	23.1 (0.8)	Male only	None	—	—	2 h	< 8	—	< 40 lx	—	21:50 (23)	—
				Blue monochrmatic LED	470 nm peak	2 h after wake	20 min	100	1344.74		—	21:20 (19)	**
				Polychromatic LED	2750 K at 250 lx	30 min before wake	50 min	0–250	106.52		—	21:38 (17)	ns

*Note:* For studies not reporting phase advances directly, we included measures comparing post‐light intervention DLMO to a dim‐light control condition. DLMO phase shift results are reported as the average phase advance in minutes (SD, minutes), while the DLMO post‐light intervention values are reported as the average DLMO timing (SD, minutes). Melanopic EDI values were calculated based on reported spectral composition data.

Abbreviations: DLMO, dim‐light melatonin onset; lx, Lux; mEDI, melanopic equivalent daylight illuminance; ns, non‐significant.

*p < 0.05

**p < 0.01.

—, not reported/relevant.

### 3.1 Light Intensity and Dose–Response Effects

A consistent finding across studies was the importance of light intensity in same‐day phase advances. For instance, Ohashi et al. [18] administered 1 h of bright light at 8000 lx shortly after waking and found a statistically significant average phase advance of 11 min. This raises the question: what is the minimum intensity required to induce a measurable phase shift? Kozaki et al. [22] addressed this issue using a within‐subjects design. Participants were exposed to 3 h of morning light at various intensities, beginning 1 h after waking. Melatonin onset was measured that same evening and compared with the previous evening's DLMO. Significant phase advances of 15, 26, and 27 min were observed for intensities of 3000, 6000, and 12 000 lx, respectively. By contrast, lower intensities of 750 and 1500 lx did not result in significant shifts.

These findings suggest the existence of a threshold or saturation effect in the dose–response relationship between the intensity of morning light and circadian phase advancement. In other words, while low‐intensity light may be insufficient to shift circadian melatonin rhythms within a single cycle, light above approximately 3000 lx appears capable of eliciting a measurable phase advance. This is consistent with other dose–response models of the non‐visual effects of light in human chronobiology, which suggest that while responsiveness increases with intensity, the effect plateaus beyond a certain threshold [23, 24]. Interestingly, to our knowledge, the relationship between dose and response for resetting human circadian melatonin rhythms by light has only been studied in relation to phase delays, rather than phase advances [25, 26].

Kozaki et al. [21] extended this work by examining the impact of morning light exposure, with the original goal of testing whether it was protective against light‐induced melatonin suppression at night. While they did not report phase shift data following the light interventions, the timing of melatonin onset advanced from dim to bright light conditions, ranging from 100 to 2700 lx. Specifically, the average timing of DLMO after dim‐light exposure was 22:15, while the average timing of DLMO after 2700 lx exposure was 21:51. Although this 24‐min average difference was not statistically significant, it suggests that even moderate‐intensity morning light may promote earlier circadian timing.

### 3.2 Light Timing and Duration

Two studies [19, 20] investigated the effects of dawn simulation, a protocol involving gradually increasing light intensity over time after waking. Although the total light intensity achieved in these studies was lower than that used in bright light protocols, the exposure lasted longer and began earlier in the circadian day.

In Gabel et al. [19], the dawn simulation began 30 min after waking. Light gradually increased from 0 to 250 lx, and this level was maintained for an additional 20 min. Participants in the comparison (dim light) condition were exposed to very low light levels throughout the same period. This relatively low‐intensity and brief exposure to the dawn simulation protocol did not result in a significant phase shift, with average DLMO timings of 21:50 and 21:38 for the dim and dawn simulation conditions, respectively. It could be argued that the low light intensity and duration levels in this dawn simulation protocol were insufficient in producing a phase‐shifting response.

By contrast, Danilenko et al. [20] conducted a more extended dawn simulation, starting 30 min after wake‐up time continuing for 2 h. This simulation reached light intensities of 1000 and 2000 lx in two separate experiments. These protocols resulted in significant phase advances of 19 and 20 min, respectively. Interestingly, these intensities are lower than those producing phase shifts in fixed morning light protocols (e.g. [22]), suggesting that longer duration and earlier timing may compensate for lower intensity. This pattern is consistent with human PRC studies, where longer‐duration light pulses (e.g., 6.7 h [10]) produce larger phase shifts than short pulses (e.g., 1 h [7]). This suggests that longer light exposure with an earlier timing in the biological morning can produce phase advances even when light intensity is moderate.

### 3.3 Spectral Composition

In addition to timing and intensity, the spectral composition of light may influence whether a phase advance can be achieved within the same cycle. Gabel et al. [19] found that 20 min of monochromatic blue light (470 nm) at just 100 photopic lux, delivered 2 h after waking, resulted in a 30‐min significantly earlier timing of DLMO (21:20 ± 19 min) compared to a dim‐light condition (21:50 ± 23 min). This finding suggests that monochromatic short‐wavelength light, even at a low intensity and duration, can produce circadian phase advances when administered in the morning. This is consistent with the well‐established spectral sensitivity of non‐image‐forming photoreception, which peaks in the short‐wavelength (blue) range [27, 28]. Thus, blue‐enriched morning light might be especially effective for inducing a same‐day phase advance.

Three out of the five reviewed studies using fixed morning light protocols employed fluorescent lighting [18, 21, 22], which typically emits less power in the short‐wavelength range compared to modern light sources such as blue LEDs or full‐spectrum light sources [29]. In fact, the reported spectral distributions in these studies confirm relatively low irradiance in the blue range, which may have reduced the effectiveness of their light interventions despite high overall lux levels. This further reinforces the importance of considering spectral characteristics of light when designing light‐induced phase‐advance interventions.

For this reason, recent guidelines [30, 31] recommend reporting light exposure using melanopic Equivalent Daylight Illuminance (mEDI), a metric that quantifies the circadian impact of light by integrating both its spectral power distribution and overall intensity. Specifically, mEDI represents the illuminance of a standard daylight spectrum (D65) that produces the same melanopic stimulation as the test light source. This biologically relevant measure offers a more accurate indication of light's potential to influence the circadian system compared to traditional photopic lux alone.

**Figure 2:**
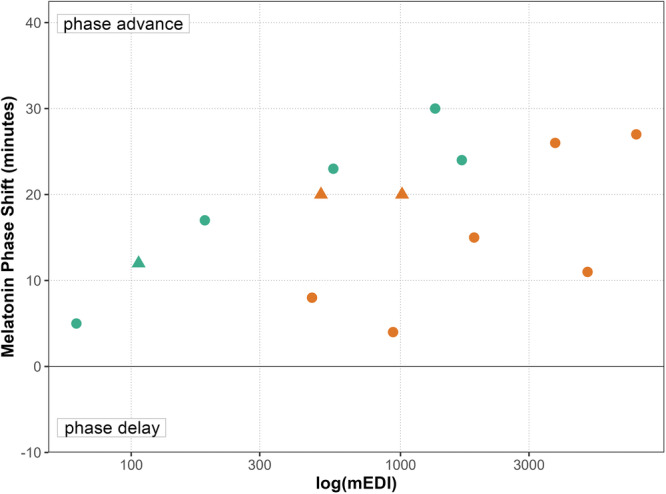
The relationship between the mEDI of the light intervention and the magnitude of the phase shift. The number of minutes of melatonin phase advance following a light intervention are reported either directly comparing a pre‐ and post‐ light intervention DLMO (orange) or compared to a dim‐light condition (cyan). Included studies involve either a light intervention protocol with a fixed light intensity (circle), or a dawn simulation protocol, where light intensity increases throughout the light intervention (triangle). Although not significant, a Spearman correlation (*r* = 0.51, *p* = 0.06, *n* = 14) indicates a trend toward a positive association between the mEDI of the light intervention and the magnitude of the phase advance. For detailed study references corresponding to each data point, see Figure S1. DLMO, dim‐light melatonin onset; mEDI, melanopic equivalent daylight illuminance.

Given the role of spectral composition and intensity in circadian phase shifting, we plotted mEDI values calculated from reported spectral data against the magnitude of phase advances observed in various studies (Figure 2). Phase advances were measured either directly (pre‐ vs. post‐light intervention DLMO) or relative to a dim‐light control condition (dim‐light DLMO vs. bright light DLMO). Overall, a Spearman correlation between mEDI and average phase advance is modest and only approaching significance, but shows a positive trend (*r* = 0.51, *p* = 0.06). This suggests that higher melanopic stimulation might generally promote greater phase advances. Interestingly, phase advances appear larger when calculated relative to a dim‐light intervention (cyan data points in Figure 2), rather than measured directly (orange data points in Figure 2). This is likely due to the intrinsic average drift of circadian rhythms during prolonged dim‐light exposure, exaggerating the magnitude of the phase advances following the light intervention.

### 3.4 Interindividual Variability

While these studies highlight the potential for same‐day phase advances, their findings also highlight a major limitation: interindividual variability. Although not all studies reported the standard deviation of the phase shift, those which did showed high variability. For instance, a significant phase average phase advance of 11 min in one study [18] showed a standard deviation of 22 min, with around one third of participants presenting a small phase delay. The standard deviations of phase shifts measured in Kozaki et al. [22] ranged from 13 to 21 min across conditions. This suggests that the effects of morning bright‐light exposure on circadian rhythms are highly variable across individuals and will not consistently cause phase shifts across all participants.

Indeed, this high interindividual variability is common in the general chronobiology literature, with non‐image‐forming light effects having been shown to be influenced by prior light history [32], caffeine intake [33], and trait factors like lens transmittance and genetics [34]. Variability in intrinsic circadian period can affect the net measured phase shift because differing amounts of endogenous drift occur between assessments, altering the apparent magnitude of light‐induced phase advances across individuals. Specifically, individuals with longer circadian period would experience a shorter net phase advance in response to morning light.

Interindividual variability in light responses is also affected by protocol design components such as non‐personalised intervention timing. For example, baseline DLMO timing in Kozaki et al. [21, 22] had a standard deviation of over an hour and their intervention was fixed at 9 a.m. Therefore, the timing of the intervention relative to DLMO will vary greatly across participants and might correspond to different regions of each individual's PRC, producing variable (and occasionally opposite) phase‐shift responses.

Taken together, the reviewed studies suggest that light‐induced circadian phase advances can occur within the same circadian cycle, particularly when bright light (> 3000 lx) is administered shortly after wake‐up time. Lower intensities may also be effective if delivered earlier (e.g., before wake through dawn simulation), or if the light is spectrally enriched in the short‐wavelength (blue) range. However, the magnitude of phase shifts is typically modest (10–30 min), and responses are highly variable across individuals, limiting the predictability of outcomes without personalised parameters.

## 4 Concluding Remarks and Open Issues

This review examined existing evidence on circadian phase advances in response to morning light exposure, with a focus on understanding when these advances occur experimentally. Is a full circadian cycle needed to observe the phase‐advancing effects of morning bright light interventions? Some studies suggest that modest, 10–30‐min circadian melatonin phase advances can be detected within the same circadian cycle as the light intervention, specifically if the light is sufficiently bright or short‐wavelength‐enriched and is timed to coincide with wake time. Nonetheless, these findings should be interpreted with caution due to limitations both in the reviewed literature and in our approach.

First, only one out of the five reviewed studies included female participants. Recent research suggests that women might be more sensitive to the circadian effects of bright light in the evening [35] and typically show an earlier chronotype than men [36]. As a result, it is unclear to what extent the results from these reviewed studies are generalisable to females: sex differences in the circadian sensitivity to morning light remain an unexplored research topic. It is also noteworthy that no examination has yet been conducted into same‐day phase advances in children, adolescents, or older adults. This remains an open avenue for investigation, considering known age‐related variations in circadian physiology and light sensitivity.

Second, not all reviewed studies adequately controlled for the post‐intervention light conditions. Given the known effects of prior light history [32, 37] and afternoon light exposure [38] on melatonin timing, not using controlled dim‐light conditions after the interventions makes it harder to isolate the specific contribution of the light stimulus to the observed phase shift.

Third, there is a lack of comprehensive experimental work in this area. Most circadian experiments measure the timing of DLMO in the subsequent circadian cycle, except the five studies discussed here that directly test same‐day phase advances. More targeted work is needed to determine whether morning light can phase‐advance human melatonin rhythms in less than 24 h, and which experimental conditions affect this.

Finally, it remains unclear whether the modest effects observed in controlled conditions would translate to occupational or clinical settings. For example, the reported same‐day phase advances might be too small to meaningfully shift melatonin timing following jet‐lag or shift work adaptation, making it important to assess their relevance in circadian interventions.

## Funding

The authors received no specific funding for this work.

## Ethics Statement

The author has nothing to report.

## Conflicts of Interest

The authors declare no conflicts of interest.

## Supporting information


**Figure S1:** The Relationship between the mEDI of the Light Intervention and the Magnitude of the Phase Advance (by author).

## Data Availability

Data sharing is not applicable as no new data was generated.
