# Supplementary material for: Can Morning Light Phase Advance Human Melatonin Rhythms in Less Than 24 h?
Source: J Pineal Res. 2026 Mar 15;78(2):e70134. doi: 10.1111/jpi.70134 (PMC12989132; doi:10.1111/jpi.70134)
Supplement: Supplementary file 1 — Figure S1: The Relationship between the mEDI of the Light Intervention and the Magnitude of the Phase Advance (by author). [file JPI-78-e70134-s001.docx]

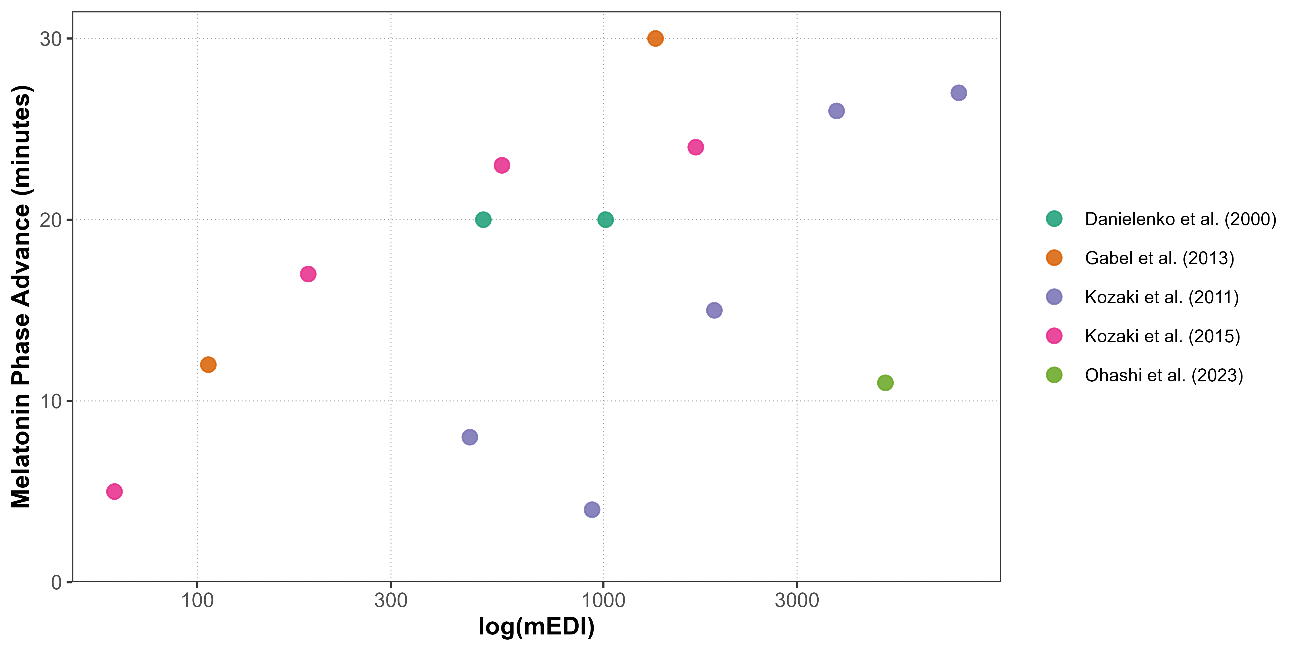


**Figure S1**

*The Relationship between the mEDI of the Light Intervention and the Magnitude of the Phase Advance (by author).*

Although non-significant, a Spearman correlation (r = 0.51, *p* = 0.06, n = 14) indicates a trend toward a positive association between the mEDI of the light intervention and the magnitude of the phase advance.

mEDI = melanopic equivalent daylight illuminance.
